# Gender-Diverse Inclusion in Immunological Research: Benefits to Science and Health

**DOI:** 10.3389/fmed.2022.909789

**Published:** 2022-07-14

**Authors:** Hannah Peckham, Kate Webb, Elizabeth C. Rosser, Gary Butler, Coziana Ciurtin

**Affiliations:** ^1^Centre for Adolescent Rheumatology Versus Arthritis at University College London (UCL), University College London Hospital (UCLH), Great Ormond Street Hospital (GOSH), London, United Kingdom; ^2^Division of Medicine, Centre for Rheumatology Research, University College London (UCL), London, United Kingdom; ^3^Department of Paediatric Rheumatology, School of Child and Adolescent Health, Red Cross War Memorial Children’s Hospital, University of Cape Town, Cape Town, South Africa; ^4^Crick African Network, The Francis Crick Institute, London, United Kingdom; ^5^Department of Paediatric and Adolescent Endocrinology, University College London Hospital (UCLH) and Great Ormond Street Institute of Child Health, University College London, London, United Kingdom; ^6^Gender Identity Development Service (GIDS), Tavistock and Portman NHS Foundation Trust, London, United Kingdom

**Keywords:** sex, gender, autoimmunity, sex hormones, sex chromosome, transgender

## Abstract

The differences between male and female immune systems are an under-researched field, ripe for discovery. This is evidenced by the stark sex biases seen in autoimmunity and infectious disease. Both the sex hormones (oestrogen and testosterone), as well as the sex chromosomes have been demonstrated to impact immune responses, in multiple ways. Historical shortcomings in reporting basic and clinical scientific findings in a sex-disaggregated manner have led not only to limited discovery of disease aetiology, but to potential inaccuracies in the estimation of the effects of diseases or interventions on females and gender-diverse groups. Here we propose not only that research subjects should include both *cis*-gender men and *cis*-gender women, but also transgender and gender-diverse people alongside them. The known interaction between the hormonal milieu and the sex chromosomes is inseparable in *cis*-gender human research, without the confounders of puberty and age. By inclusion of those pursuing hormonal affirmation of their gender identity- the individual and interactive investigation of hormones and chromosomes is permitted. Not only does this allow for a fine-tuned dissection of these individual effects, but it allows for discovery that is both pertinent and relevant to a far wider portion of the population. There is an unmet need for detailed treatment follow-up of the transgender community- little is known of the potential benefits and risks of hormonal supplementation on the immune system, nor indeed on many other health and disease outcomes. Our research team has pioneered the inclusion of gender-diverse persons in our basic research in adolescent autoimmune rheumatic diseases. We review here the many avenues that remain unexplored, and suggest ways in which other groups and teams can broaden their horizons and invest in a future for medicine that is both fruitful and inclusive.

## Introduction

The pertinent sex bias in the human immune system is a phenomenon that may never have come to light, were it not for significant policy changes that enforced the inclusion of female participants alongside males in medical research (1). Historically, clinical trials were conducted predominantly on male subjects only, or failed to discriminate between outcomes experienced by males vs. females (2). Justified by pragmatic reasons, predominantly healthy young males were recruited to avoid potential toxicity risks associated with pregnancy and breastfeeding, while excluding more mature patients of both sexes to decrease the risk of concomitant comorbidity. Little differed in basic scientific research, where male-only mouse models mitigated the outcome variability potentially resulting from the menstrual cycle or pregnancy, and most *in vitro* human work failed to report the sex of the cell lines used (3). This approach is not only inaccurate in answering research questions relevant to humans, irrespective of sex and gender, but is also potentially harmful in underestimating the effects of interventions on females and other gender-diverse groups. Although medical understanding and subsequent research study design have advanced significantly in recent years, this chronic failure to recognise the importance of sex as a key biological variable has by no means been fully overcome. Anecdotally, in attempting to collect data on global COVID-19 morbidity and mortality between the sexes, it was notable how few countries or local authorities were reliably disaggregating their outcome statistics according to patients’ sex, even at later stages of the pandemic (4). Sophisticated national platforms detailed deaths according to geographical regions, age groups and occupational categories, but frequently neglected to mention sex. Our meta-analysis (5), alongside several other studies (6–8), showed a significant male bias in severe outcomes and deaths from SARS-CoV-2; a pattern mirrored in the vast majority of infectious diseases (9–11) and variously suggested to relate to sex hormone levels (12–14). The enhanced ability of the female immune system to clear invading pathogens is further supported by its ability to mount generally stronger responses to most vaccinations (15–17). For example, in adults given the seasonal Trivalent Inactivated Influenza Vaccine, female responses to a half-dose were comparable to those of males given a full-dose (18). The inverse of this is of course the female predisposition to developing autoimmune disorders associated with a hyper-active immune system, such as systemic lupus erythematosus (SLE), where the male:female ratio is estimated at 4–13:1, according to different studies (19–28).

Both hormonal and chromosomal factors are suggested to contribute to immunological sex differences. Oestradiol is broadly thought of as immunostimulatory, with testosterone having a more regulatory effect (29), though both have demonstrated either capability, as reviewed elsewhere (30–33). Meanwhile the X chromosome encodes the most immune-related genes of any chromosome (34) such as TLR7 [toll like receptor, responsible for sensing viral and endogenous nucleic acids to trigger release of type 1 interferons, and implicated in extrafollicular B cell class switch recombination (35)], CD40-L [co-stimulatory T cell molecule, essential for B cell class switching (36)], FoxP3 [controls regulatory T cells (37)] and CXCR3 [chemokine receptor, recruits effector T cells to sites of inflammation (38)]. This is highlighted by the abundance of X-linked immune disorders such as immunodysregulation polyendocrinopathy enteropathy X-linked (or IPEX) syndrome, X-linked agammaglobulinemia and Wiskott Aldrich Syndrome, which are associated with cellular and humoral immune deficiencies and increased risk of infections from childhood (39). Several immune genes on the X chromosome may escape the X-inactivation of one chromosome in 46,XX individuals, and thus be bi-allelically expressed, potentially resulting in altered immune regulation (40–43). Whilst studies have sought to investigate the contributions of hormonal and/or chromosomal influences on the immune response, it is recognised that it is a complex nexus and mutual interaction of the two that ultimately leads to such notable sex biases in infection and autoimmunity. With this in mind, this review seeks to highlight the importance of including subjects of both sexes, as well as transgender people in immunological research, to enable evaluation of sex-biased clinical outcomes and provide benefit to our understanding of the biology of the immune system with relevance for both science and health.

## Gender Identities and Physical Phenotypes

**FIGURE 1:**
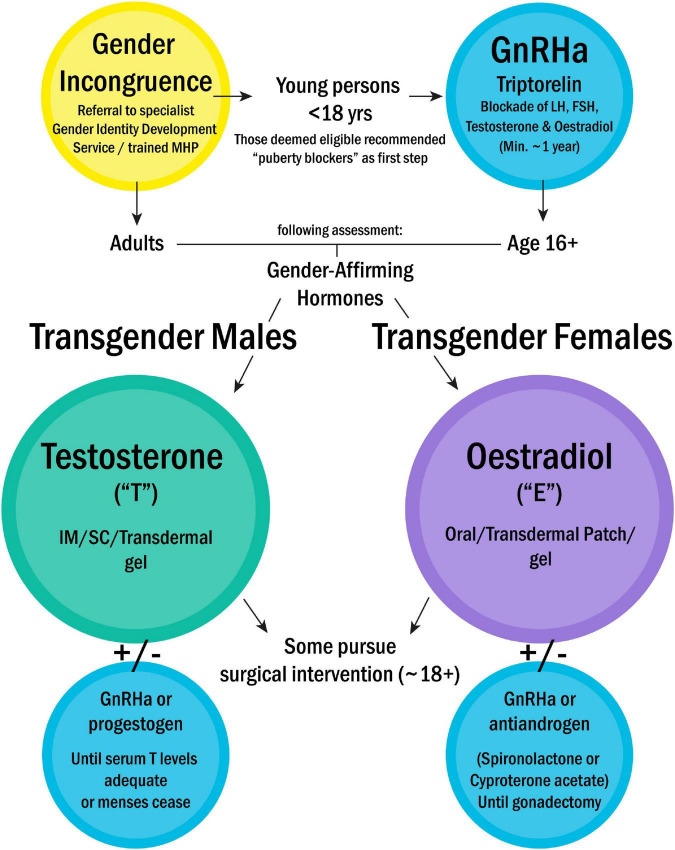
Treatment pathway for gender incongruence, as recommended by the Endocrine Society (42). Treatment is prescribed on a case-by-case basis, based on individual country guidelines. This flowchart outlines the most commonly pursued routes. NB- Parenteral oestradiol not currently used in Europe. *MHP, Mental health professional; GnRHa, Gonadotropin releasing hormone analogs; LH, Luteinising hormone; FSH, Follicle stimulating hormone; IM, Intramuscular; SC, Subcutaneous.*

For the majority of the population, the terms sex and gender describe the binary categories of “cisgender male” and “cisgender female”; with experienced gender matching the sex registered at birth, which is itself based upon simple observation of the genitalia of the new-born baby. Frequently assimilated within the category of “other,” however, are a multitude of gender identities and physical phenotypes. By “transgender” we refer broadly to those whose experienced gender identity does *not* match that in which they were registered at birth. Thus, trans-males, are registered female at birth, typically carry a 46,XX chromosomal background, and may pursue virilisation *via* testosterone treatment and/or oestradiol blockade. Trans-females, are registered male at birth, typically of 46,XY chromosomal background, and may pursue gender-affirming oestradiol treatment and/or testosterone blockade (44). Specific treatment pathways and medications recommended by the Endocrine Society (45) are summarised in Figure 1. A third main category are those who are non-binary/gender fluid (not identifying exclusively and/or permanently as either gender); some of whom may seek hormonal blockade *via* treatments such as the gonadotropin releasing hormone analogs (GnRHa), or specific hormonal blockades. There is also the category of differences/disorders of sex development (previously known as ‘intersex’), where people may have physical characteristics of both sexes (gonadal structures, genitalia) and this umbrella term also includes those with karyotype variations of sex development such as Klinefelter syndrome [47,XXY] and Turner syndrome [45,X] (46). Lastly but by no means exhaustively are those classified as “agender”- not identifying with any gender at all. Many other gender-related groupings exist, beyond the scope of this review, but we have included here the main categories pertinent to immunological research.

To refer again to international COVID statistics, even fewer countries reported outcomes in those who were not cisgender. In some countries, the catch-all ‘other’ category was reported alongside cisgender males and females; but this was representative of so many diverse groups that granular analyses of differential gender-related outcomes could not be possible. Such is the case for the vast majority of outcome reporting in health and disease, suggesting that better characterisation of populations pertaining to self-reported gender is warranted. In the United Kingdom alone, referrals to the NHS young people’s Gender Identity Development Service (GIDS) have increased by over 2000% in the last 10 years (47); this represents a growing proportion of society who are frequently not even adequately recognised in statistics, let alone included in basic science or relevant clinical research. Here we examine potential ways in which inclusion of a broader spectrum of gender groups can improve our scientific understanding of the pathogenesis of both infectious diseases and autoimmune disorders, as well as providing potentially pertinent clinical information for under-represented groups and the physicians involved in their care.

The multitude of gender-related social factors that may contribute to increased vulnerability to different medical conditions are beyond the scope of this paper and reviewed elsewhere (48). However, the physiological impact of a person’s sex chromosomal makeup combined with their hormonal milieu (be this endogenous or medically supplemented) is what we propose to be an important focus of future research. In *cis*-gender people, the contributions of sex chromosomes and hormones are inextricably linked. We know both to be of significance, but researchers currently are able to separate these factors to examine how they interact and separately contribute only in animal models and *in vitro* research. By inclusion of trans or gender-diverse persons pursuing hormonal affirmation of their gender, we are able to investigate the effects of hormonal manipulation on the immune system in healthy individuals of a wide age range (usually older than 16 in the United Kingdom).

## Sex Bias in the Epidemiology and Outcomes of Autoimmune Rheumatic Diseases

The majority of autoimmune rheumatic disorders (ARDs) affect *cis*-females in greater number than *cis*-males, as is the case with SLE, Sjögren’s syndrome (SS) (49), scleroderma (SSc) (50) and rheumatoid arthritis (RA) (51). SLE predominantly affects females of child-bearing age, with incidence pre-puberty significantly lower (52) and pregnancy associated with increased flares in patients with recently active disease (53, 54). Taken together, these epidemiological observations strongly suggest a role for the sex hormones in disease pathogenesis. However, juvenile rheumatic diseases, defined as having onset before the age of 16–18 years depending on phenotype, such as juvenile idiopathic arthritis (JIA), juvenile lupus (JSLE), juvenile Sjögren’s syndrome (JSS) and juvenile dermatomyositis (JDM) *also* exhibit sex bias, but this is less prominent than in their corresponding adult-onset phenotypes (55). JIA, for example, has no significant sex bias overall as an umbrella term, but different disease sub-types are characterised by different age at onset and sex-predominance: e.g., Enthesitis Related Arthritis (ERA) affects predominantly boys and has onset around puberty, while subtypes oligo- and poly-arthritis are more common in pre-pubertal and post-pubertal girls, respectively (56). As pre-pubertal *cis*-boys and *cis*-girls have similar serum sex hormone levels, a potential role for the sex chromosomes in the disease pathogenesis is thus also supported.

Several studies have investigated the effect of hormonal medications in SLE, where one might expect to see exacerbation of disease upon use of the oral contraceptive (OC), or hormone replacement therapy with oestradiol (HRT) given to alleviate menopausal symptoms. Commonly cited is the *Nurse Health Study*, which followed thousands of ciswomen, and reported an elevated relative risk for the development of SLE of 1.9 for women who had ever used hormonal OC (57) and of 2.1 in post-menopausal women who had ever used (HRT) (58). Although hormonal treatments have been purported to cause flares in SLE in older studies (59), recent literature has demonstrated little to no impact of OC usage on mild to moderate SLE, with the potential for unplanned pregnancies deemed a more significant risk for patients than OC use (60, 61). Several studies have demonstrated reduced androgen levels in SLE patients (62, 63), and this has been suggested to play a role in disease development or severity. Therein, the use of various forms of androgen as therapeutic agents has been tested in several incidences – with some trials showing mild efficacy (64–68) while others showed no difference from placebo (69). Thus, the current literature on *in vivo* manipulation of hormones does not provide a conclusive picture. Several case studies (70–77) detail the development of autoimmunity in trans-females upon commencement of gender-affirming oestradiol treatment, or the improvement of symptoms when taking gender-affirming testosterone (78). However, one cannot infer causality from these instances, nor can individual case studies be extrapolated to the wider population. Inclusion of trans people in bigger cohort studies on autoimmunity development is thus strongly supported – whether the increased relative risk seen in post-menopausal *cis*-females on HRT would be the same or similar in trans-women with an XY chromosomal background is yet unknown.

Although the majority of autoimmune diseases are characterised by female bias, there is evidence that type I diabetes mellitus and Crohn’s disease are characterised by a male predominance, irrespective of age at onset (79, 80). Additionally, some conditions have differential disease phenotypes according to sex, which has implications in disease recognition and epidemiological data collection. This is the case with spondyloarthritis (81), which had been considered a male-predominant disease for many decades before evidence about a different clinical presentation and delays in diagnosing females with spondyloarthritis emerged (82). Further, certain treatments may be more efficacious in one sex compared to the other [recently reviewed extensively by Klein and Morgan (83)], e.g., TNF inhibitors tend to work better for males with RA than for females (84) and female patients may be more likely to stop such drugs following the side effects they experience from them (85). Moreover, there is evidence that spontaneous puberty can completely reverse the sex bias in disorders of immune regulation such as asthma and atopy, characterised by male preponderance prepuberty, followed by a significantly increased female prevalence during reproductive years (86).

## Impact of Age, Puberty and Menopause on Autoimmunity

Throughout the various life stages from infancy to old age, the immune system is also subject to great change (87, 88), and these changes are known to differ between cisgender males and females (89). The ageing immune system is a growing area of research, but less is known specifically about the immune changes that may occur during/after puberty and menopause. The coincidence of the average age of onset of several juvenile rheumatic diseases (90) with the average age of puberty onset (91) suggests that it is not merely the maturation process itself that alters one’s immune system, but that the rise in sex hormone levels seen in puberty is also involved. Our systematic review of the bidirectional relationship between puberty and autoimmune rheumatic disorders demonstrated how poorly these relationships are documented in the literature, but highlighted the differences in disease outcome in those with onset pre- vs. post-puberty (92) and symptomatic differences have been noted between different age groups of SLE patients (93), with adolescent onset JSLE noted for its greater severity (94, 95). In the case of menopause, RA (96) and SSc (97) both have their peak incidence in the over 50 age bracket. SLE has classically been considered to have its peak incidence within the childbearing years in females, but a 10-year incidence study of United Kingdom patients found the peak onset to be between 50 and 54 years in females and 70–74 in males (98), and this was supported by two other shorter studies (21, 99). However, these studies were of predominantly white populations, and in studies including black (100), Arab (101) and American Indian (102) patients, younger ages of peak onset between 30.4 and 39.2 have been observed. It is unclear exactly why this might be, but this highlights the complexity of sex-based influences on the immune system, which may interact with both age- and ethnicity-related factors to give rise to autoimmunity. With the inclusion of transgender subjects of different ages and pubertal/menopausal stages among basic and clinical research, these factors could be separated out, and the impact of sex be examined without the confounders of immunosenescence and ethnically inherited risk factors.

## Differential Effect of Sex Determinants on Immune Activation Pathways

The investigation of the impact of sex-determinants on certain immune activation pathways, such as specific cell populations or pro-inflammatory pathways, where both sex chromosomal and hormonal elements have been separately suggested to be of influence is an area with great scope for new discovery. Work from our lab, published in 2019 (103), pioneered the inclusion of gender-diverse cohorts to address questions relevant to SLE, using a cohort of healthy trans- (*n* = 13 male, 7 female) and cisgender (*n* = 48 male, 51 female) young volunteers, alongside individuals with Turner Syndrome (*n* = 9), who are missing an X chromosome (45,X). Young transgender healthy controls were recruited from the University College London Hospital GIDS and treatment pathways are shown in Figure 1. Production of the antiviral cytokine family known as type 1 interferons (IFN)- predominantly by plasmacytoid dendritic cells (pDC)- is known to contribute significantly to the pathogenesis of both SLE and JSLE. We demonstrated that pDC from healthy *cis*-females produced more T1 IFN in response to TLR-7 signalling than pDC from *cis*-males, even before puberty. Using our inclusive volunteer cohort, we were additionally able to show that this related to X chromosome dosage *and* serum testosterone concentration, in a manner that was dependent upon the number of X chromosomes present. Overall, we showed that both factors were associated not just individually, but also interactively with the T1 IFN response.

More recently, we used a similar cohort (*n* = 17 *cis*-male; 22 *cis*-female; 10 trans-male and 10 trans-female) to examine the effects of sex and hormones on regulatory and responder CD4 + T cells (Tregs and Tresps, respectively) (104). Sex differences in Tregs are well-reported (105–109), and we firstly confirmed the observation that healthy *cis*-males have higher levels of Tregs compared to Tresps than *cis*-females both pre- and post-puberty. We then demonstrated that the ability of *cis*-male Tregs to suppress the division of Tresps was significantly enhanced compared to that of *cis*-female Tregs, supporting the concept of a pro-inflammatory phenotype in females that could contribute to autoimmunity. Then, using RNA sequencing (RNAseq), we found a significant number of differentially expressed genes (DEGs) in sorted Tregs from *cis*-males compared to females. Using our transgender healthy controls, we observed significant differences in related immune pathways following hormone treatment, demonstrating the potential for both oestradiol and testosterone to impact Tregs at a transcriptional level, even at the early stages of their treatment.

The COVID-19 pandemic has prompted several interesting studies on sex differences in viral responses, and how these translate into clinical outcomes. Takahashi et al. (8) demonstrated a more robust T cell response in females with the disease, compared to males- with poor T cell responses associated with a worse disease trajectory in males. Meanwhile males had higher levels of innate inflammatory cytokines, but higher levels of these in females were associated with more severe outcomes. Supporting these findings, Liu et al. (110) compared transcriptional differences in healthy males and females, demonstrating that males had higher expression of proinflammatory cytokines and chemokines, which they hypothesise may contribute to the ‘cytokine storm’ that is detrimental in COVID-19 pathogenesis. Females in this study were found to have higher expression of IFN genes, supporting what is already known about the sex bias in IFN production in health and in autoimmunity. These data demonstrate a clear link between sexual dimorphism in the immunological systems that serve to protect us, that may also lead to damage in the context of an autoimmune disease. Inclusion of trans and gender-diverse cohorts in infection response studies, is thus equally warranted alongside those in autoimmunity.

There remain myriad of cell types and mechanisms that have been identified as potentially influenced by sex hormones or chromosomes, thus meriting *in vivo* interrogation. In addition to the further work necessitated on pDCs, the T1 IFN pathway, and Tregs/Tresps, obvious suggestions for future research directions (based on preliminary evidence of sex hormonal/chromosomal effect in animal or non-diverse cohorts) are B cells and antibody/autoantibody production (111–120), B regulatory (Breg) cells (121), CD4 T cells (116, 122–124), and specific T helper subsets (89, 125–131), CD8 cytotoxic T cells (122, 132–135), dendritic cells (136–140), Natural Killer (NK) cells (116, 141–145), neutrophils (146–149), monocytes (150) and macrophages (149, 151, 152). Table 1 summarises a selection of notable effects of sex determinants on immune processes and cell types known to be relevant to autoimmune rheumatic disease- this is by no means an exhaustive review of the literature, and many extensive reviews are available (89, 182, 183). As a field in its relative infancy, there remain so many avenues ripe for gender-disaggregated interrogation and scintillating project proposals.

**TABLE 1:** Summary of notable immune system elements known to be regulated by sex determinants and their relevance to autoimmune rheumatic disease.

	*Cis*-female	*Cis*-male	Relevance to autoimmune rheumatic diseases (ARD)
**Immune cells**			
B cells	Oestrogens shown to: alter the threshold for B cell apoptosis/activation (112); increase capacity for class-switch recombination (114, 115, 117, 113, 119).	Androgens act *via* GPR174 to divert B cells from germinal centre formation and subsequent class-switching (120). Testosterone regulates *BAFF* – important in survival of autoreactive B cells (118).	Production of autoantibodies central to pathogenesis of many ARDs.
*Immunoglobulins*	Higher plasma Ig levels in females (111, 116).	–	
CD8 T cells	Lower cell frequency but higher cytotoxic capacity in females (135).	Higher cell frequencies in males (122, 123, 132).	Multiple roles across ARDs (153, 154).
CD4 T cells	Higher cell frequencies in females (116, 122, 123, 132).	–	Subset imbalance (155) and functional abnormalities in SLE (156). Pathogenic role in JIA uveitis (157).
*Treg subset*	Androgens enhance female CD4 + T cell FoxP3 expression *in vitro* (158).	Male Tregs had greater suppressive ability (104).	Impaired immune regulation in SLE and RA (125).
*Th17 subset*	Oestrogens both stimulatory (126, 127) and suppressive (128) of proliferation and IL-17 production. Activation *via* ERβ enhances Th17 response, *via* ERα suppresses (130).	Frequency of IL-17A and Th17 cells increased in males with AS compared to females with AS (129).	Role in SLE disease manifestations (159) and IL-17 in RA (160). Initiation of SS (161). Th17 axis implicated in AS pathology (162).
*Th1 subset*	Oestrogen and progesterone decrease Th1:Th2 and Th17:Th2 cytokine production ratios (131). Male V female Th1 or Th2 predominance varies, reviewed in (89).		SS initiation (Th1) and progression (Th2) Psianou et al. (161) Th1:Th2 imbalance in RA (163).
*Th2 subset*			
Macrophages and Monocytes	Macrophage phagocytic activity higher in females (146).	Testosterone increases monocyte counts in men (149).	Inflammatory damage to cartilage and bone in RA etc. (164). Defects in phagocytosis and clearance of cellular debris in SLE (165).
Dendritic Cells (DC)	E2 enhanced ability of DCs to activate CD4 + Th cells *in vitro* (136, 138).	Higher levels in hypogonadic males inversely correlated to testosterone levels (140).	Presentation of self-antigen.
*Plasmacytoid Dendritic Cells (pDC)*	More activated in females and produce more IFN-α (103, 166).	–	IFN production prominent role in SLE pathogenesis (167).
Neutrophils	Phagocytic activity higher in females (146). Oestrogens and progesterone can affect lifespan (147) and numbers increased during luteal phase of menstruation and in pregnancy (148).	Testosterone increases counts in men (149).	Release of proinflammatory cytokines and NET formation externalises autoantigens (168).
Natural Killer Cells (NK)	Higher cell number in females (154). Progesterone contributes to accumulation during pregnancy (144).	Increased CNS NK inflammation in males vs. females in ALS mouse model- NK depletion benefitted females but not males (145).	Cytotoxicity in inflammation and role in immunoregulation/immune homeostasis (169).
**Cytokines** and **Immune Mediators**			
Type 1 Interferons	IFN-α production higher in female cells post TLR stimulation (103, 170).	Testosterone correlates with IFN-α independently from X chromosome (103).	Prominent role in SLE pathogenesis (167).
Type 2 Interferons	E2 treatment in mice increased DC production of IFN-γ (138).	IFN-γ higher in stimulated lymphocyte supernatant from males (170).	Inflammatory role in SLE, SS, SSc and dermatomyositis (171).
IL-10	Higher production in stimulated lymphocyte supernatant from females (170).	Higher production in males and correlates with testosterone (172).	Breg and IL-10 role in SLE, RA and SSC (173).
Microbiota	Bi-directional relationship between hormones and microbiota, with immune impact (174, 175).		Known impact of microbiota on rheumatic disorders (176).
**Transcriptional Differences**			
Macrophages (MF)	Higher expression of MF IFN-stimulated genes in female mice, with sig. bias in antiviral response genes (177).	–	IFN role in SLE, SS, SSc, RA and dermatomyositis (178).
CD8 Cytotoxic cells	Greater toxicity post-stimulation in female cells: antiviral and inflammatory gene exp increased, many with oestrogen response elements in their promoters (134).	–	Multiple roles across ARDs (155, 156).
*AIRE* (autoimmune regulator) expression	Oestrogens inhibit (179).	Androgens enhance (180).	Necessary for self-tolerance induction in the thymus (181).

*BAFF, B cell Activating Factor; Ig, Immunoglobulins; ERα, Oestrogen Receptor Alpha; ERβ, Oestrogen Receptor Beta; AS, Ankylosing Spondylitis; CNS, Central Nervous System; ALS, Amyotrophic Lateral Sclerosis; IFN, Interferon; TLR, Toll-like Receptor.*

## Unanswered Questions and Future Directions

There is an unmet need for better understanding of the long-term outcomes of sex hormone manipulation on the health of trans and gender-diverse people. This includes the effects of gender-affirming treatment on responses to natural and vaccine immunisations, on bone and muscle health, as well as their impact on mental health and quality of life, before moving into investigating infective and autoimmunity risk in these populations. Without accurate gender classifications in population studies, these relevant outcomes cannot be studied. There are many specific questions which need answering in relation to the impact of sex determinants on immune system functions, in particular around exposure to and timings of exposure to sex hormones. We do not know if the length of exposure to/blockade of a particular sex hormone is different from the physiological sex hormone fluctuations, especially those related to menstruation, pregnancy, or early stages of puberty/menopause. There is no research into the impact of age at which a person is first exposed to (or begins blocking) sex hormones on their risk of infections, autoimmunity, or other adverse health outcomes. Our group identified a significant impact of sex hormones in driving a pro-atherogenic lipid profile in healthy *cis*- and *trans*-male adolescents post-puberty (184). Therefore, investigating the impact of sex-affirming hormone therapy on the cardio-vascular risk of trans people has a clear clinical rationale. Further research is needed to investigate the effects of lifetime exposure to higher exogenous oestrogen or androgen therapies, especially in the context of potential reversibility and dose-dependent long-term effects. In some countries, young people are able to commence puberty blockade and gender-affirming sex hormones prior to the commencement of their natural puberty. Meanwhile in the United Kingdom, only those aged 16 + and thus likely already post-pubertal can legally be consented to start on gender-affirming hormone treatments. Others still, may not access treatment until much later into adulthood. It is important to establish whether outcomes (immunological or otherwise) would be similar or different in these groups, when their hormonal transitions have commenced at such widespread life stages. Furthermore, it is possible that different routes of hormone administration (oral, patch, gel, IM, SC.) and dosages of these may impact the systems of the human body differently. Innovative clinical trial study design, including volunteers of all gender categories, across various age ranges is required to be able to examine the relative importance of sex hormone exposure at different stages of life, against both sex chromosomal backgrounds, on various interventions or health and disease outcomes. In addition, the inclusion of subjects with altered sex chromosomal complement (such as Klinefelter and Turner syndromes) could provide suitable controls for these studies aiming to tease out the distinct effects of various sex chromosome determinants.

**FIGURE 2:**
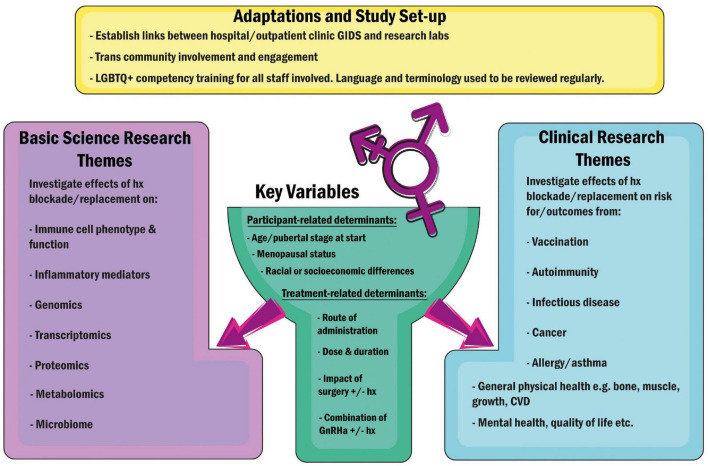
Suggested adaptations to facilitate future research encompassing trans and gender-diverse individuals, and key research pathways proposed. *Hx, Hormones; GnRHa, Gonadotropin Releasing Hormone Agonists (“Blockers”); CVD, Cardiovascular Disease.*

First steps would be establishing national and international registries with associated biological sample repositories capturing patients of various gender categories, sex chromosomal backgrounds and demographic diversity to enable long-term follow-up. A number of social barriers exist, well-documented in the United States, that prevent the trans population from accessing healthcare and thus participating in research (185). Thus, it is important for such registries to be set up with advice and input from transgender charities and organisations such as *WPATH* (World Professional Association for Transgender Health) on how to overcome these barriers. This should include ensuring that all health professionals and researchers involved are trained in LGBTQ + cultural competency (186), so that all elements of study design- from language used on questionnaires, to subtlety when approaching people for recruitment- are optimised to help participants feel secure and respected. Further, recruitment must extend beyond private healthcare patients, encompassing public healthcare clinics as well as community support groups, in order to capture the true breadth of the trans population. Hospital and clinic record databases must be updated in order to capture gender definitions and associated medications more accurately, and reference ranges for clinical and laboratory tests need to be reviewed and established for gender-diverse people, as it is highly likely that they may differ from those appropriate for *cis* persons (187). If these changes were made across the world, they would not only facilitate far more impactful retrospective review of outcomes, but would vastly improve the lives and healthcare of transgender persons, who have tolerated systems that weren’t designed to accommodate them for far too long. In Figure 2, we propose several streams of research, both clinical and immunological, as starting points for future projects. Researchers and clinicians should join forces to give people of all gender identities a voice and create opportunities for their involvement in clinical data collection and research. As more countries develop their gender identity services, and adapt to the changes outlined above, we look forward to seeing the results from further large studies such as 2021 *Michelson Prize* recipient Dr. Camila Consiglio’s multi-parameter analysis of the effect of testosterone treatment on the immune systems of trans-men at the *Karolinska Institutet*, Sweden (188), and that of Professor Guy T’Sjoen’s *ENIGI* consortium across Ghent, Oslo, Florence, and Amsterdam (189, 190), where long-term follow-up of participants pursuing hormonal gender affirmation will provide us with a wealth of information, pertinent to everyone – not just those it is convenient to study.

## Concluding Remarks

We advocate that research should celebrate gender diversity and be as inclusive as possible to ensure that it is relevant to human society as a whole. We can only hope that in coming years, more labs and clinical teams will join us in the interrogation of sex determinants as biological variables. As personalised medicine becomes an increasingly viable and beneficial approach to healthcare, it is research like this that will be equipped to inform and steer innovation in the appropriate direction.

## Disclaimer

Gender-related terminology is continually evolving, and terms vary in their usage between individuals and between groups across the world. Language and definitions used throughout this article have been adapted from the Gender Identity Research and Education Society (GIRES) website at time of writing (191) – we have made every effort to be inclusive, but acknowledge that these may not capture the preferences and experiences of all.

## Author Contributions

CC, GB, and HP contributed to conception of the review. HP wrote the first draft of the manuscript and designed the figures. CC, GB, KW, and ER wrote sections of the manuscript. All authors contributed to manuscript revision, read, and approved the submitted version.

## Conflict of Interest

The authors declare that the research was conducted in the absence of any commercial or financial relationships that could be construed as a potential conflict of interest.

## Publisher’s Note

All claims expressed in this article are solely those of the authors and do not necessarily represent those of their affiliated organizations, or those of the publisher, the editors and the reviewers. Any product that may be evaluated in this article, or claim that may be made by its manufacturer, is not guaranteed or endorsed by the publisher.
